# Mechanism of thrombocytopenia in COVID-19 patients

**DOI:** 10.1007/s00277-020-04019-0

**Published:** 2020-04-15

**Authors:** Panyang Xu, Qi Zhou, Jiancheng Xu

**Affiliations:** 1 Department of Laboratory Medicine, First Hospital of Jilin University, Changchun, 130021 China; 2 Department of Pediatrics, First Hospital of Jilin University, Changchun, 130021 China

**Keywords:** Severe acute respiratory syndrome coronavirus 2, Coronavirus disease 2019, Thrombocytopenia, Platelet

## Abstract

Since December 2019, a novel coronavirus has spread throughout China and across the world, causing a continuous increase in confirmed cases within a short period of time. Some studies reported cases of thrombocytopenia, but hardly any studies mentioned how the virus causes thrombocytopenia. We propose several mechanisms by which coronavirus disease 2019 causes thrombocytopenia to better understand this disease and provide more clinical treatment options.

## Introduction

Since December 2019, many patients with coronavirus disease 2019 (COVID-19) pneumonia have been discovered in Wuhan, Hubei province, China. This virus subsequently spread to other provinces in China, and patients have been discovered in other countries [1–3]. Novel coronavirus pneumonia is a novel respiratory disease in humans that is caused by the novel coronavirus. The WHO has officially named this disease coronavirus disease 2019 (COVID-19). Currently, six coronaviruses that can infect humans have been discovered (HCoV-229E, HCoV-OC43, HCoV-NL63, HCoV-HKU1, SARS-CoV, and MERS-CoV). The first four viruses mainly cause the common cold, whereas the SARS-CoV and MERS-CoV viruses cause severe acute respiratory syndrome (SARS) and Middle East respiratory syndrome (MERS), respectively. The newly discovered coronavirus is a β-coronavirus that has enveloped virus particles that are spherical or oval in shape. Although it belongs to the same genus as SARS-CoV and MERS-CoV, its genetic characteristics show significant differences compared with SARS-CoV and MERS-CoV [4]. After assessment of the virus, the Coronavirus Study Group of the International Committee on Virus Taxonomy recommended naming this virus severe acute respiratory syndrome coronavirus 2 (SARS-CoV-2). The epidemiological data provided by Huang et al. showed that the Huanan Seafood Wholesale Market in Wuhan was the source of the zoonosis. The appearance of disease clusters proved that human-to-human transmission is present [5]. Some researchers found that the full-length genome sequence of SARS-CoV-2 obtained from earlier patients had a homology of 79.5% with the SARS-CoV sequence and a homology of 96% with the whole genome of bat coronaviruses [6]. This provided valuable clues for examining the pathogenesis and clinical treatment of COVID-19.

## Thrombocytopenia in patients with COVID-19

**Fig. 1:**
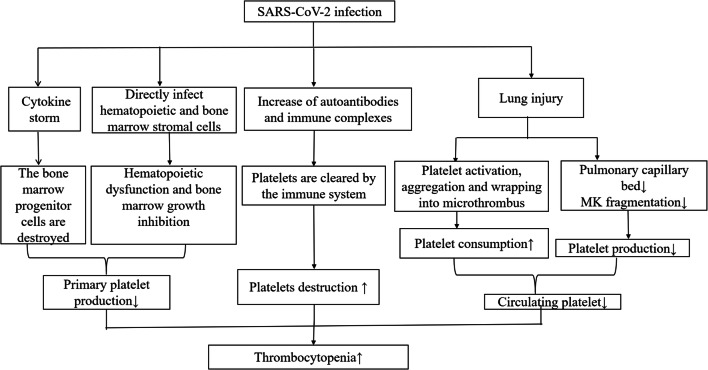
The possible mechanisms of thrombocytopenia in COVID-19 patients. SARS-CoV-2, severe acute respiratory syndrome coronavirus 2; COVID-19, coronavirus disease 2019; MK, megakaryocyte;↑, means an increase in a substance; ↓, means a decrease in a substance

The most common symptoms seen in COVID-19 patients are fever, fatigue, and dry cough, and dyspnea gradually develops. Some patients have mild symptoms at disease onset and may not present with apparent fever. Uncommon symptoms include abdominal pain, headache, palpitations, and chest pain. Hematological changes are common in patients with COVID-19, which include reduced lymphocyte count and platelet count but normal white blood cell count. Prolonged activated partial thromboplastin time, 26% had elevated D-dimer levels, and most patients had normal prothrombin time (PT) [7]. Of seven patients in the University of Hong Kong-Shenzhen Hospital (Shenzhen, Guangdong province, China), two had thrombocytopenia, and two had elevated D-dimer levels [8]. A study involving 1099 patients from 31 provinces/direct-controlled municipalities in China showed that 82.1% of patients had lymphopenia, 36.2% had thrombocytopenia, and 33.7% had leukopenia [9]. These laboratory marker abnormalities were more significant in severe cases [9]. In 13 patients from 3 hospitals in Beijing, 72.5% developed thrombocytopenia [10]. Statistics from 41 patients in a designated hospital in Wuhan showed that 5% of patients had thrombocytopenia on admission [11]. In most cases, the platelet count did not decrease to a level at which bleeding occurs. However, the mechanisms by which this coronavirus interferes with the hematopoietic system are unclear. In this paper, we summarized the hematological changes of thrombocytopenia in patients with COVID-19 and proposed possible mechanisms by which COVID-19 causes thrombocytopenia (Fig. 1).

## The possible mechanism of thrombocytopenia in COVID-19

### SARS-CoV-2 may reduce platelet production

Coronaviruses are able to infect bone marrow cells, resulting in abnormal hematopoiesis [12]. SARS-CoV-2 and human SARS-CoV have 82% nucleotide homology [13]. Because SARS-CoV and HCoV-229E have identical antigen characteristics, it is speculated that SARS-CoV-2 and HCoV-229E antigens have some similarity. Human aminopeptidase N (CD13) is a metalloprotease that is present on the cell surfaces of epithelial cells in the intestine, kidneys, and lungs and is a receptor for HCoV-229E [14]. CD13 is a marker of granulocytes and monocytes and is ubiquitous in respiratory tract epithelial cells, smooth muscle cells, fibroblasts, epithelial cells in the kidneys and small intestine, activated endothelial cells, lymphocytes, and platelets. HCoV-229E enters bone marrow cells and platelets through CD13 receptors and induces growth inhibition and apoptosis in the bone marrow, leading to aberrant hematopoiesis and thrombocytopenia [14]. Thrombocytopenia caused by SARS-CoV-2 infection is similar to that caused by SARS-CoV and HCoV-229E infection. Based on this phenomenon, it is speculated that SARS-CoV-2 similarly inhibits hematopoiesis in the bone marrow through certain receptors to cause decreased primary platelet formation and lead to thrombocytopenia.

Secondary hemophagocytic lymphohistiocytosis (sHLH) is caused by excessive proliferation and activation of mononuclear macrophage system, in which a large number of inflammatory cytokines are released and a large number of blood cells are swallowed. This reactive disease has a rapid response with high mortality, and its basic features include persistent fever, hyperferremia, cytopenia, and lung involvement. In the retrospective analysis of 150 patients with COVID-19 in Wuhan, China, it was found that elevated ferritin was one of the predictors of death [15]. After analyzing the blood samples of 33 severe and critical type ill COVID-19 patients, Wei Haiming’s team found that after novel coronavirus infection, T cells were overactivated to produce granulocyte-macrophage colony-stimulating factor (GM-CSF) and interleukin-6 (IL-6). GM-CSF stimulated CD14+CD16+, inflammatory mononuclear macrophages to produce more interleukin-6 (IL-6), and other inflammatory factors, thus forming an inflammatory storm and causing immune damage to the lungs and other organs [16]. This is similar to the clinical manifestation and laboratory examination of patients with sHLH. In addition, studies have shown [15] that the cytokine spectrum similar to sHLH is related to the severity of COVID-19 disease. It is speculated that after the cytokine storm, the hematopoietic progenitor cells in bone marrow of patients with pneumonia infected by novel coronavirus were destroyed, the primary production of platelets decreased, and at the same time, too many blood cells were swallowed, which led to the decrease of peripheral blood platelet count.

Evidence [17] has shown that a large number of megakaryocytes dynamically release platelets during pulmonary circulation. Persistent hypertension and oxygen toxicity exacerbate lung injury, resulting in consolidation changes such as fibrosis. Damaged pulmonary capillary beds cause the process of megakaryocyte rupture and platelet release to be blocked, which affects the release of platelets into the pulmonary circulation and indirectly leads to reduced platelet synthesis in the systemic circulation.

### SARS-CoV-2 infection may increase platelet destruction

COVID-19 may increase levels of autoantibodies and immune complexes, resulting in specific destruction of platelets by the immune system. A study reported that the phenomenon of immune-mediated thrombocytopenia in patients infected with HIV-1 is widespread [18]. Although the pathogenesis is unknown, this was proven to be associated with circulating immune complexes containing platelet membrane components and the anti-platelet membrane GPIIIa49-66 IgG antibodies [18]. Anti-platelet membrane GPIIIa49-66 IgG antibodies can cross-react with the HIV-1GP 160/120 antigen. Antibodies and immune complexes deposited on the surfaces of platelets will be recognized by reticuloendothelial cells, and the platelets will be destroyed as target tissues, resulting in excessive platelet destruction. Platelets with similar antigens may be coated by anti-platelet antibodies and immune complexes, which may result in immune-mediated damage. In addition, antibodies produced during viral infection may specifically bind to antigens on platelets through molecular mimicry, resulting in increased platelet destruction.

### SARS-CoV-2 infection may increase platelet consumption

Viral infection and inflammation result in lung damage. Damaged lung tissues and pulmonary endothelial cells may activate platelets in the lungs, resulting in aggregation and formation of microthrombi, which increases platelet consumption. Most patients with COVID-19 who have thrombocytopenia have elevated D-dimer levels and impaired coagulation time, which further proves the above hypothesis that there is low intravascular coagulation. Therefore, it is still unclear which drugs used for the treatment of patients with COVID-19 having thrombocytopenia resulted in recovery. SARS-CoV-2, MERS-CoV, and SARS-CoV are all β-coronaviruses. Previously, a patient with MERS received large doses of corticosteroids by intravenous infusion to treat thrombocytopenia, and their platelet counts improved [19]. This classical method has been shown to correct thrombocytopenia in patients infected with HIV [20]. Therefore, it is speculated that the intravenous injection of human immunoglobulin, corticosteroids, and platelets may be effective in patients under certain circumstances. In the treatment measures of Diagnosis and Treatment Protocol for COVID-19 (Trial Version 7) [21], it is pointed out that for patients with excessive activation of inflammatory response, the recommended dose of glucocorticoid can be used in a short period of time. This is consistent with the above studies to improve the thrombocytopenia. Reverse transcriptase inhibitors are effective in the treatment of HIV-related thrombocytopenia. For example, zidovudine increased platelet synthesis. In addition, drug stimulation of megakaryocyte synthesis can increase platelet synthesis. Evidence shows that the chemokine CXCR4 can be expressed in megakaryocytes. Because SARS-CoV-2 and HIV are both RNA viruses, reverse transcriptase inhibitors and chemokine receptor antagonists may improve the disease course of COVID-19. At the same time, it is suggested that Shenmai injection can be used to treat immunosuppression in the treatment of traditional Chinese medicine in Diagnosis and Treatment Protocol for COVID-19 (Trial Version 7) [21]. Shenmai injection has a scavenging effect on all kinds of pathological substances. It can improve anticoagulation and thrombocytopenia in patients with COVID-19 effectively. Additionally, the immunotherapy scheme of “monoclonal antibody drug topirazumab + routine therapy” is also included in the Diagnosis and Treatment Protocol for COVID-19 (Trial Version 7) [21] as an valid option treating severe and critical COVID-19 cases. The monoclonal antibody against IL-6 receptor tocilizumab can effectively block COVID-19’s inflammatory storm, thus improving the prognosis of the patients.

## Summary

There are very few reports of the mechanisms of thrombocytopenia in patients with COVID-19, but thrombocytopenia is very common. Through analogy, three mechanisms of thrombocytopenia are hypothesized in this review:

1. Direct infection of bone marrow cells by the virus and inhibition of platelet synthesis. Following virus infection, cytokine storm destroys bone marrow progenitor cells and leads to the decrease of platelet production. Lung injury indirectly results in reduction of platelet synthesis.
2. Platelet destruction by the immune system.
3. Platelet aggregation in the lungs, resulting in microthrombi and platelet consumption.

Further investigation of the mechanisms of thrombocytopenia can provide a valuable theoretical basis for timely clinical treatment and provide us with a more comprehensive understanding of this disease.
